# The Delay of *Raphanus raphanistrum* subsp. *sativus* (L.) Domin Seed Germination Induced by Coumarin Is Mediated by a Lower Ability to Sustain the Energetic Metabolism

**DOI:** 10.3390/plants11070843

**Published:** 2022-03-22

**Authors:** Fabrizio Araniti, Bhakti Prinsi, Luca Espen

**Affiliations:** Dipartimento di Scienze Agrarie e Ambientali—Produzione, Territorio, Agroenergia, Università Statale di Milano, Via Celoria n° 2, 20133 Milano, Italy; bhakti.prinsi@unimi.it (B.P.); luca.espen@unimi.it (L.E.)

**Keywords:** phytotoxicity, seed germination, respiration, plant metabolism, ^31^P-NMR

## Abstract

In the present study, the mode of action of coumarin using the germination process as a target was investigated. A dose–response curve, built using a range of concentrations from 0 to 800 µM, allowed us to identify a key concentration (400 µM) inhibiting the germination process, reducing its speed without compromising seed development. Successively, short time-course (0–48 h) experiments were carried out to evaluate the biochemical and metabolic processes involved in coumarin-induced germination delay. The results pointed out that coumarin delayed K^+^, Ca^2+^, and Mg^2+^ reabsorption, suggesting a late membrane reorganisation. Similarly, seed respiration was inhibited during the first 24 h but recovered after 48 h. Those results agreed with ATP levels, which followed the same trend. In addition, the untargeted metabolomic analysis allowed to identify, among the pathways significantly impacted by the treatment, amino acids metabolism, the TCA cycle, and the glyoxylate pathway. The results highlighted that coumarin was able to interact with membranes reorganisation, delaying them and reducing the production of ATP, as also supported by pathway analysis and cell respiration. The in vivo ^31^P-NMR analysis supported the hypothesis that the concentration chosen was able to affect plant metabolism, maintaining, on the other hand, its viability, which is extremely important for studying natural compounds’ mode of action.

## 1. Introduction

The study of natural compounds with phytotoxic activity to develop new weed-management strategies is a challenging task due to the complexity of their mode of action and their potential multi-target activity [1]. Specialised metabolites’ biosynthesis is intrinsically connected with the evolutionary forces that drive species’ evolution and their survival strategies adopted in response to biotic and abiotic stresses in specific ecosystems. This ecological pressure led to new specialised metabolic pathways usable by plants for their defence and adaptation, which produce a wide array of structurally different chemicals with high biological activity [2]. This phenomenon has been compared by Dayan and Duke [2] to a high-throughput screen where scientists test several compounds over a brief period on a single biochemical target. However, natural processes selected specialised metabolites over millions of years based on their biological activities on whole plants and involved several chemical interactions with countless organisms and target sites. Nowadays, more than 2,140,000 specialised metabolites have been isolated and are classified according to their vast diversity in biosynthesis, structure, and function [3]. Few of them have been examined for their phytotoxic potential, and the modes of action (MOAs) of even fewer have been elucidated [4].

Among the different classes of specialised metabolites, hydroxycinnamic acids and their derivatives are highly potent natural phytotoxins [5]. In particular, one of the most biologically active classes of molecules is the coumarins class, deriving from the lactonisation of the o-hydroxycinnamic acid. The simplest compound representing this class is coumarin (also known as 1,2-benzopyrone), studied mainly for its inhibitory ability on seed germination and seedlings’ growth [6,7,8,9,10,11,12]. It has recently been demonstrated that coumarin’s phytotoxic effects on seedling growth are due to an accumulation of cyclin B, which alters the root apical meristem architecture mining the integrity of microtubule cortical array organisation. Such alterations are related to a reduction in auxin basipetal transport to the apical root meristem and its accumulation in the maturation zone, stimulating lateral root formation [13]. Despite the vast amount of studies focused on seedlings’ growth and metabolism in response to coumarin [14,15], only a few manuscripts tried to elucidate its effects on seed germination.

The inhibition or delay of seed germination is an important effect that plays a significant ecological role in natural ecosystems, increasing the competitive ability of the species that can retard the growth of the competitors. McCalla and Norstard [16] defined germination as the vegetative stage most sensitive to phytotoxins. In fact, a short period of inhibition or stimulation, at this stage, could strongly increase or reduce the ability of the seedling to compete with other plants [17,18]. The plants’ ability to delay the germination of neighbouring species through the release of coumarin in soil was largely documented, even if only a few hints were suggested concerning its mode of action [19].

One of the first studies on coumarin’s mode of action was reported by Nutile [20], which described, for the first time, the inhibitory activity of coumarin on the germination of lettuce seeds, connecting it to the induction of light-sensitive dormancy in lettuce seeds. Mayer and Evenari [21], studying the coumarin structure–activity relationship, reported that the germination inhibitory activity was due to its specific structure (an unsaturated lactone linked to an unsubstituted benzene nucleus), but they also added that any change in coumarin structure caused a slight reduction, but not a destruction, of its activity as germination inhibitor. Successively, Mayer and Poljakoff-Mayber [22] reported that although it did not prevent the breakdown of sucrose, coumarin did prevent the accumulation of glucose. At the same time, a block in lipase activity was observed, suggesting that coumarin might operate primarily as an enzyme inhibitor with no marked specificity, but no studies confirmed this hypothesis. It was also suggested that the coumarin-induced dormancy in lettuce seeds could be attributable to its ability to antagonise gibberellins’ function, since the exogenous addition of these hormones was unable to revert its effects [23,24].

More recent research highlighted the role of a seed coat in mediating the inhibitory activity of coumarin on radish germination; since, as a result of the tegument removal, the molecule’s biological activity was significantly reduced, it was suggested that coumarin could induce a secondary seed dormancy, according to Aliotta, Fuggi, and Strumia [12]. In addition, they demonstrated that coumarin (200 µM) significantly inhibited radish germination by 50%, and that this molecule inhibits the elongation of cells of the differentiating zone of the root produced by seeds germinated in the treatment.

One of the earliest studies on specific enzymes involved in durum wheat seed germination was published by Abenavoli et al. [9], who reported that coumarin delayed the reactivation of peroxidases, enhanced the superoxide dismutase activity, and reduced the activity of selected marker enzymes involved in metabolic resumption. Later, Pergo et al. [6] suggested that coumarin acts as a cytostatic agent, retarding germination and growth of *Bidens pilosa* and exerting, at higher concentrations, stimulation of lipoxygenase activity. Chen et al. [8], working on the embryo of rice seeds, observed that coumarin (in a range between 1 mM and 20 mM) delays germination by decreasing the ABA 8′-hydroxylase 2 and 3 genes (OsABA8′ox2/3), inhibiting, as a consequence, the catabolism of abscisic acid. In addition, in explaining the role of endogenous hormone and their interaction with coumarin during germination, recent studies highlighted that this specialised bioactive metabolite might delay seed germination by reducing endogenous GA4 and decreasing, as a consequence, the accumulation of ROS [7].

Till now, experiments focused on germination response to coumarin were carried out on seeds treated for several days with the molecule at significantly high concentrations. Considering the peculiar events that characterise the first phase of seed germination, such as membrane repair and reorganisation, activation of different metabolic pathways, and the induction of macromolecules biosynthesis, to analyze coumarin’s effects in the first instances of germination appear crucial to better investigate the mechanisms underlying its toxicity. Therefore, to highlight the early effects of coumarin on this sensitive physiological process, we decided to work with a short time course, using the molecule at a sublethal concentration and carrying out in vivo (classical physiological and biochemical methods joined to ^31^P-NMR experiments) and destructive untargeted metabolomics experiments. These approaches allowed to describe the metabolic changes induced by coumarin treatment on radish seed germination, highlighting the pathways affected by its inhibitory activity.

## 2. Results

### 2.1. Germination Index and Seed Respiration

**Figure 1:**
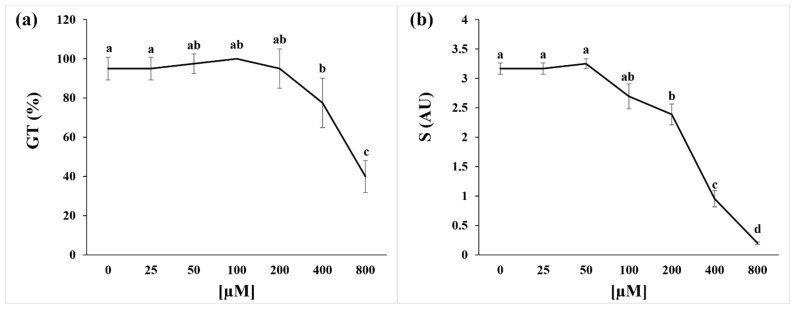
Dose–response curves of the (**a**) germination index (GT(%)) and (**b**) germination speed (S; Arbitrary Unit, AU) of *R. sativus* seeds treated for 48 h with 0–800 µM of coumarin. Data are expressed as mean ± SD and analysed through one way ANOVA using the LSD test as post hoc (*p* ≤ 0.05). Different letters along the bars indicate statistical differences among the treatments. N = 4.

The dose–response curve showed that coumarin treatment significantly affected the GT index at concentrations higher than 200 µM, inducing inhibition of 18% and 58% at 400 and 800 µM, respectively (Figure 1a). On the contrary, the S parameter highlighted a significant reduction in speed germination already at a concentration of 200 µM (25% lower than control), which reached 70% inhibition at 400 µM and 90% at 800 µM (Figure 1b).

Since 400 µM was the first significant concentration able to inhibit seed germination and delay this process, we decided to focus all the following experiments using this concentration.

### 2.2. Ion Leakage and Reabsorption and Seed Respiration

**Figure 2:**
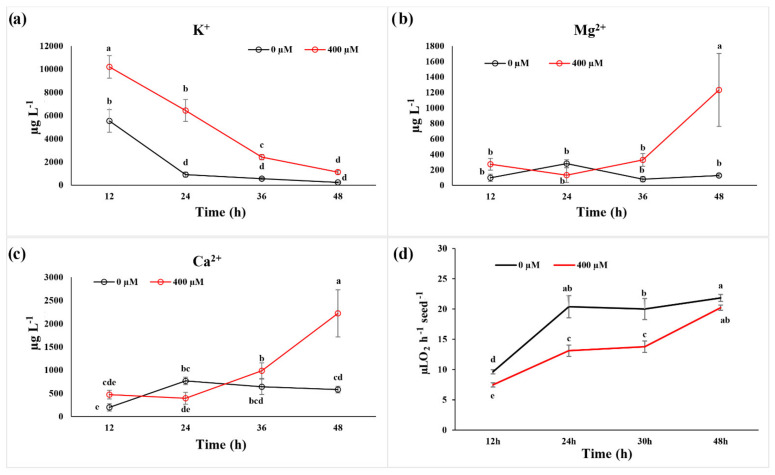
Effect of coumarin (400 µM) on levels of (**a**) K^+^; (**b**) Mg^2+^ and (**c**) Ca^2+^ in the incubation medium during the first 48 h of seed germination in *R. sativus*. (**d**) Time-course monitoring of the effects of coumarin (400 µM) on seed respiration. Data are the means + SD and analysed through two-way ANOVA using the LSD test as post hoc (*p* ≤ 0.05). Different letters along the curves indicate statistical differences among the treatments. N = 3.

The ion leakage, monitored on the external medium in which seeds were incubated, highlighted significant differences in Ca^2+^ and Mg^2+^ only after 48 h of treatment, at which nine-fold higher Mg^2+^ and 4-fold higher Ca^2+^ content in the external medium were observed (Figure 2b,c). On the contrary, K^+^ content was higher than the control at 12 h (one-fold), 24 h (seven-fold), and 36 h (four-fold) of treatment, but reached control values after 48 h (Figure 2a). 

Concerning seed respiration, evaluated on control and treated seeds (400 µM) in a time-course experiment (6–48 h), no changes were observed after 6 h of treatment (data not shown), whereas 12 h and 24 h of exposition reduced this parameter by 36% and 30%, respectively (Figure 2d). On the contrary, no differences were observed after 48 h, where an almost complete recovery was observed in the treated seeds (Figure 2d).

### 2.3. In Vivo NMR Analysis

**Figure 3:**
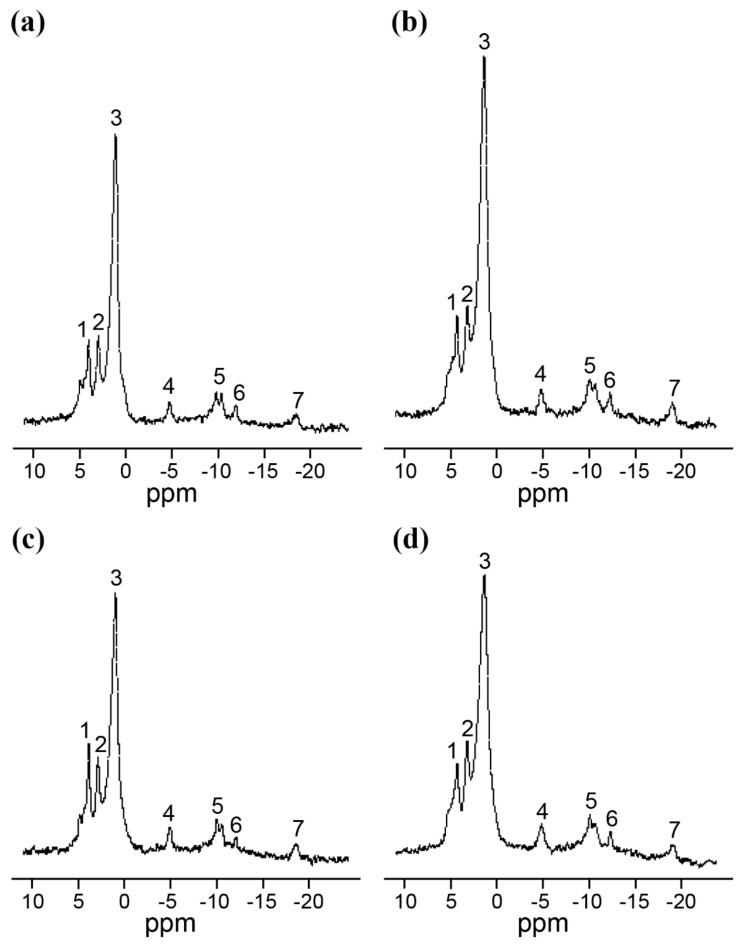
Representative ^31^P-NMR spectra of *R. sativus* seeds incubated for 24 (**c**,**d**) and 48 h (**a**,**b**) in the absence (**a**,**c**) or in the presence (**b**,**d**) of 400 µM of coumarin. The spectra were acquired with a 1 s recycle time and are the sum of 2000 scans. The resonance assignments are as follows: peak 1, phosphocholine; peak 2, cytoplasmic inorganic phosphate; peak 3, vacuolar inorganic phosphate; peak 4, γ-phosphate of NTP and β-phosphate of nucleoside diphosphate (NDP); peak 5, α-phosphates of NTP and NDP; peak 8, UDP-Glc and NAD(P)(H); peak 6, UDP-Glc; peak 7, β-phosphate of NTP. Chemical shifts are quoted relative to 85% H_3_PO_4_. In the three independent experiments, the resonance intensities differed by 15% at most.

The in vivo ^31^P-NMR analysis carried out on seedlings germinated on coumarin (0 µM and 400 µM) for 24 h and 48 h pointed out significant differences among the spectra and the parameters evaluated (Figure 3). However, from the metabolic point of view, all spectra showed the resolution (i.e., the presence of peaks referable to main *p*-metabolites and the possibility to discriminate the inorganic phosphate pools, corresponding to those present in the cytoplasm and vacuole, respectively) typical of an active tissue, allowing us to exclude general toxicity symptoms in the seeds incubated in 400 µM of coumarin.

Concerning the cytoplasmatic pH, no differences were observed after 24 h and 48 h, whereas after 48 h of treatment, a reduction in the vacuolar pH was observed in control plants (Table 1).

**Figure 4:**
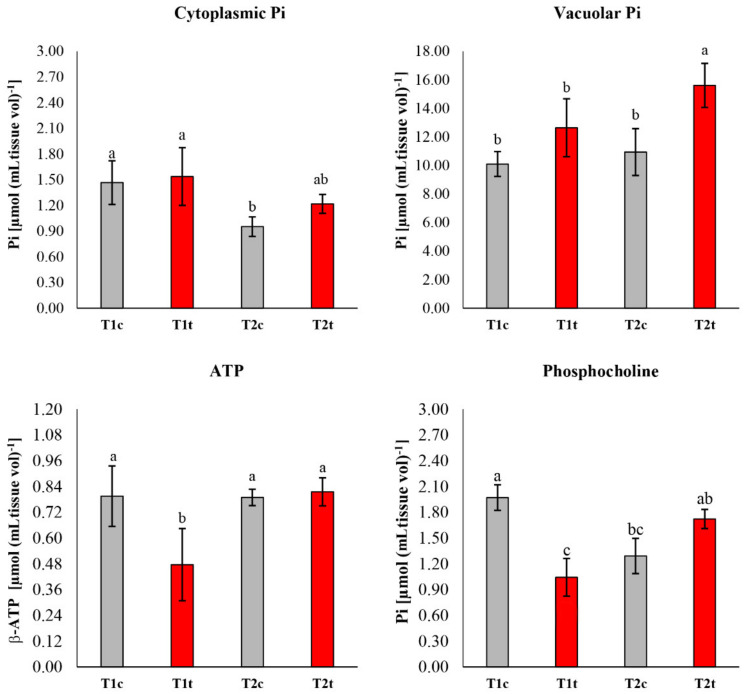
^31^P-NMR study of the in vivo effects of coumarin treatment (400 µM for 24 h and 48 h) on *R. sativus* seed germination monitoring cytoplasmic and vacuolar phosphate, ATP, and phosphocholine content. T1c (control, 24 h), T2c (control, 48 h), T1t (treated, 24 h), T2t (treated, 48 h). Data are expressed as mean ± SD. Data were analysed through two-way ANOVA using the LSD test as post hoc (*p* ≤ 0.05). Different letters along the bars indicate statistical differences among the treatments. N = 3.

A significant reduction in the amount of cytoplasmatic inorganic phosphate was observed in control seedlings after 48 h (Figure 4). On the contrary, the vacuolar inorganic phosphate was significantly higher in seedlings treated with coumarin for 48 h (Figure 4). Concerning ATP content (Figure 4), coumarin-treated seedlings were characterised by a lower content after 24 h of treatment and a general recovery to the control level after 48 h. A similar trend was also observed in phosphocholine content, which was significantly lower after 24 h of treatment and recovered to the control level at the end of the experiment (48 h) (Figure 4).

### 2.4. GC-MS Untargeted Metabolomic

To obtain more insights into coumarin-induced metabolic changes in *R. sativus* seeds during germination, a GC/MS-driven untargeted-metabolomic analysis was carried out.

The GC-MS-driven analysis was performed on seeds treated with coumarin for 0 (T0), 24 (T1), and 48 h (T2). The analysis revealed grouped and individual metabolites that allowed samples’ discrimination. Among all the analysed stages, the metabolomic analysis allowed us to annotate and quantify 72 metabolites (mainly primary metabolites) and extract 161 unknown EI-MS shared features. Successively, the unknown features were putatively annotated in silico through MS-Finder, which allowed us to annotate the putative structure of 73 metabolites (mainly specialised metabolites).

Unsupervised Principal Component Analysis (PCA) was carried out on blank samples and the two sample groups (treated and untreated from T0 to T2) to demonstrate the system suitability (Figure S1a–d). The PCA Score Plot, built on the first (PC1) and the second component (PC2), revealed good discrimination of sample groups against blanks, highlighting model robustness (Figure S1a). The components used allowed the separation of the two treatments and the three growth stages (T0–T2) with no outliers (Figure S1a–d), indicating that our metabolomic analysis was reliable and could sufficiently reflect the metabolic profile changes of the seeds during germination. The unsupervised PCA, run on MS-DIAL-suggested metabolites (Figure S1c) and unknown features (Figure S1d), revealed clear discrimination of sample groups.

**Figure 5:**
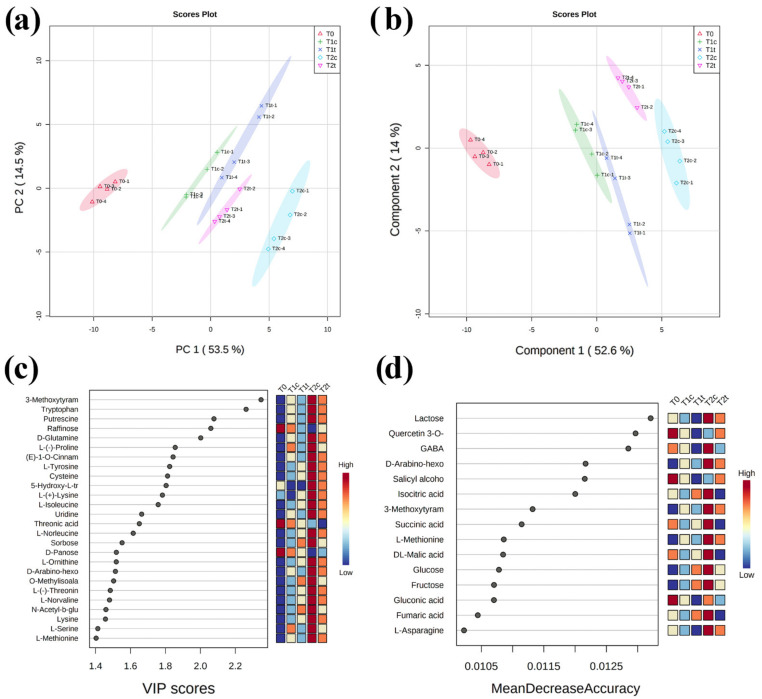
Discrimination through principal component analysis (PCA) and partial least-squares discriminant analysis (PLS-DA) of the metabolites’ patterns in *R. sativus* seeds exposed for 0 h, 24 h, and 48 h to 0 µM and 400 µM of coumarin. T0 (untreated seeds at the beginning of the experiments), T1c (control, 24 h), T2c (control, 48 h), T1t (treated, 24 h), T2t (treated, 48 h). (**a**) PCA and (**b**) PLS-DA showing score plots that allowed groups discrimination by virtue of the first two principal components (PCs). (**c**) Variable importance of projection (VIP) features for the groups from PLS-DA analysis. (**d**) Random forest analysis displaying the mean decrease in accuracies. (N = 4).

Successively, after manual feature annotation (on both MS-DIAL and MS-Finder) and the discard of falsely annotated metabolites, the putative metabolites and their normalised intensities were analysed through Metaboanalyst 5.0 [25]. The unsupervised PCA carried out on annotated metabolites was performed to obtain a global view of the time-course changes in metabolic patterns of developing *R. sativus* seeds during coumarin treatment (Figure 5a). The first principal component (PC1), accounting for 53.5% of the total variance (PC2 accounted for 14.5%), reflected time-dependent seed germination development in response to coumarin. Clear separation among times and treatments was observed (Figure 5a).

The evaluation of the PCA loading plots pointed out that the PC1 was mainly dominated by 3-methoxytyramine-betaxanthin, tryptophan, D-glutamine, L-(-)-proline, and raffinose, among others (Table S1), whereas PC2 was mainly dominated by specialised metabolites such as 5-hydroxy-L-tryptophan, eremopetasitenin A1, gentiflavine, and solanapyrone, among others (Table S1).

To obtain maximal covariance between the metabolite levels and the treatments at each time point, a partial least-squares discriminant analysis (PLS-DA) was applied (Figure 5b). The PLS-DA model was well described using the first four components (Figure S2), which explained a total variance higher than 75% (Figure S2). Moreover, the cross-validation and permutation test validated the PLS-DA model’s robustness, highlighting a high R2 and Q2 for both latent variables and a *p* ≤ 0.05 (Figure S3). PLS-DA derived variable importance in projection (VIP) scores (built on the first 30 metabolites with a VIP score higher than 1.4) revealed 3-metoxytyramine BX, tryptophane, putrescine, raffinose, D-glutamine, proline, among others, like the ones with the highest VIP scores for the three germination steps (Figure 5c). Finally, a random forest analysis revealed lactose, quercetin-3-O-glucuronide, GABA, D-Arabino-hexose-2-ulose, and salicin, among others, as the metabolites with the highest mean decrease in accuracy (features ranked by their contributions to classification accuracy) for the three sample groups (Figure 5d).

**Figure 6:**
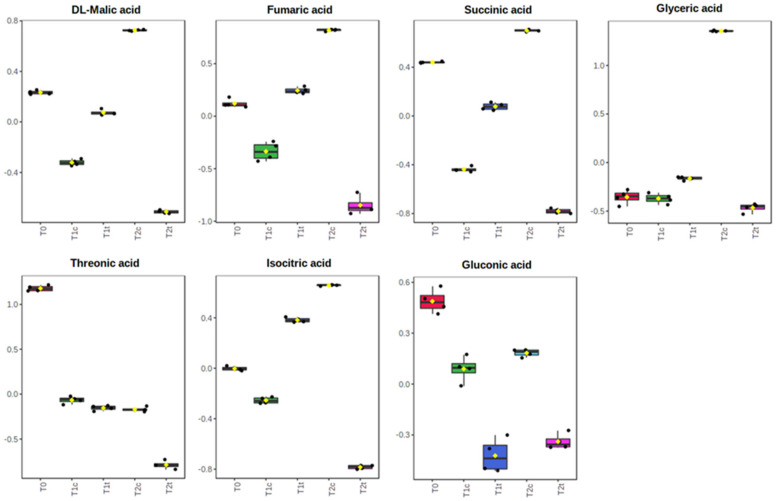
Effects of 0 h, 24 h, and 48 h of coumarin treatment (400 µM) on the contents in *R. sativus* seeds from the organic acids pool. Normalised metabolomic data were analysed through ANOVA using the LSD test as post hoc (*p* ≤ 0.05). N = 4. For the full list of the significantly altered metabolites, refer to Supplementary Table S1.

**Figure 7:**
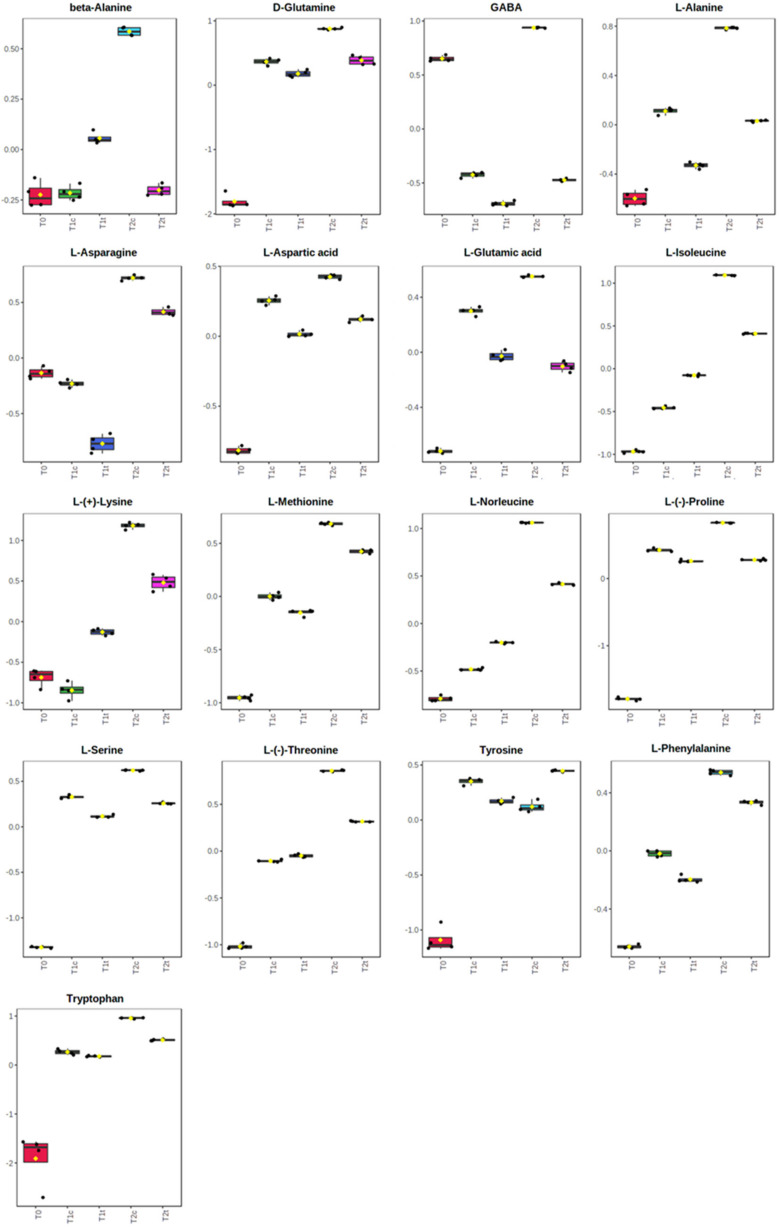
Effects of 0 h, 24 h, and 48 h of coumarin treatment (400 µM) on the contents in *R. sativus* seeds from amino acids pool. Normalised metabolomic data were analysed through ANOVA using the LSD test as post hoc (*p* ≤ 0.05). N = 4. For the full list of the significantly altered metabolites, refer to Supplementary Table S1.

The univariate analysis highlighted 126 significantly altered metabolites among the three times of exposure and the two concentrations assayed (for the full list of the significantly altered metabolites, refer to Supplementary Table S1). Among them, the organic acids (Figure 6) were generally in higher abundance than the control after 24 h of treatment but then significantly dropped after 48 h (Figure 6). On the contrary, the abundance of the majority of the amino acids significantly increased compared to T0 after 24 h (T1) and 48 h (T2) of treatment, but their content was, in general, lower in treated plants than in untreated plants (Figure 7). Attention should be paid to lysine, for which peculiar behaviour was pointed out. Indeed, the treated seeds showed a higher level after 24 h but a lower one after 48 h compared to control seeds (Figure 7).

A KEGG-based pathway analysis, which combines enrichment and topology analysis, was carried out separately, comparing coumarin’s effect at T1 and T2. The analysis revealed that 29 (at T1) and 35 pathways (at T2) were significantly altered by the treatment (Table 2 and Supplementary Table S1). However, at T1, only 22, and at T2, only 25 were characterised by an impact higher than 0.1 (Table 2 and Supplementary Table S1). Among them, amino acids metabolism, the glyoxylate pathway, and the TCA cycle, all playing pivotal roles during seed germination, were significantly impacted by coumarin treatment (Table 2).

## 3. Discussion

Identifying the mode of action (MoA) of a natural compound is a challenging task that requires biochemical and physiological knowledge as well as the choice of the right concentration and the right time of exposure to the chemical [1]. The choice of the effective concentration is generally based on standard parameters such as IC_50_ and LD_50_, among others [26]. At the same time, the main problem encountered during the study of a molecule’s MoA is the choice of lengthy exposures to the chemical, which do not allow the observation of the primary effects on plant metabolism but only the side effects, mainly due to a cascade of biochemical and physiological responses to the stressing factor. In the present study, focused on coumarin effects on seed germination, we used horseradish (*R. sativus*) as the target species since it is considered a sensitive species for the phytotoxicity tests. Moreover, it is characterised by a quick and synchronised germination (~24 h), which is essential to reduce samples variability, and is known to be sensitive—during germination—to coumarin exposition [10,11,12]. In addition, before the experiments, the seeds were decoated to avoid the seed-coat-mediated dormancy already observed during coumarin treatments [12]. Concerning the choice of the concentration, we focused our attention on the lowest effective dose that inhibited the germination process and delayed its speed. In particular, we focused our study on the concentration of 400 µM that—compared to other old and recent studies, which analysed the effects of coumarin at concentrations ≥1 mM [8,9,27]—could be considered a low dose. For this study, we decided to combine classical physiological methods, such as respiration monitoring, with in vivo (^31^P-NMR) and destructive (GC-MS-driven untargeted metabolomics) metabolomics techniques in order to monitor metabolic changes in response to the induced stress.

The dose–response curve built on germination data confirmed the effective phytotoxicity of this molecule, which was in agreement with previous studies carried out on the same species [28] at concentrations ≥ than 400 µM. However, the reduction in this parameter was already evident at 200 µM. During the first stages of the germination process, the seed is a disorganised structure that must reorganise its membranes before activating the biochemical processes that lead to energy production, cell division, and root protrusion [29]. During this reorganisation stage, several ions, such as K^+^, Ca^2+^, and Mg^2+^, are leaked by the seeds and then reabsorbed after the membrane reorganisation [30,31]. Our results suggest that coumarin affected the plasma membrane reorganisation and the reactivation of transport activities, delaying ions reabsorption. In particular, in coumarin presence, after an early (12 h and 24 h) increase in K^+^ release in the germination medium, K^+^ was reabsorbed at the same rate as in control seeds. On the contrary, lesser reabsorption of Mg^2+^ and Ca^2+^ was observed after 48 h of treatment. Similar effects (K^+^ reabsorption and Mg^2+^ leakage) were observed on radish seeds treated with the phytotoxic concentrations of nickel, which did not alter plasma membrane reorganisation but was only delayed ion reabsorption, slowing down the germination process [32]. This effect was attributed to the lesser availability of energy to sustain transport activities [32]. Considering that it was recently highlighted that treatment with Ca^2+^ mobilisation inhibitors hindered radicle protrusion in rice (Oryza sativa) seeds [33], the observed Ca^2+^ accumulation in the germination medium and the delay of its reabsorption could suggest a slower reactivation of germination processes in coumarin-treated seeds. The central role of calcium in the metabolic reactivation is also well-supported by the study conducted by Negrini and co-workers [34]. In that work it was demonstrated that the reduction in Ca^2+^ availability, obtained by the addition of Na-EGTA into the incubation medium, negatively affected the activation of the calmodulin pathway (i.e., the formation of a Ca-calmodulin complex), and this effect is strictly related to a delay in the radish seed germination. In conclusion, the specific effect of coumarin on Ca^2+^ reabsorption, which was not observed for K^+^, could represent a crucial point in its inhibitory action. Phosphocoline is an intermediate in phospholipids’ biosynthesis that is related to membrane biosynthesis, a crucial event in seed germination [35,36]. In the present study, phosphocholine, with production that is strictly dependent on ATP, was significantly lower after 24 h of treatments (in agreement with the lower ATP level observed) but was restored to the control level after 48 h. These data strongly support the hypothesis that during the first 24 h of treatment, coumarin could delay membrane synthesis and organisation. It has been reported that in A. thaliana, the abundance of phosphatidic acid, mediated by phosphatidic acid phosphohydrolase activity, modulates CTP:phosphocholine cytidylyltransferase activity to govern phosphocholine content [37].

To support the hypothesis that coumarin did not alter seed reorganisation, but it was only delaying it, we decided to monitor the seed respiration, which was already known to be altered by coumarin treatment on the root [38], but barely described on seed germination. The data pointed out no differences in seed respiration after 6 h of coumarin exposure (data not shown), and this is expected since the biochemical activities of the seeds during the first stages of germination are extremely low, and the main sources of gas release are from colloidal or simply pushed out from gas-filled spaces by the water that is rushing in [39]. On the contrary, significant lower O_2_ consumption was observed between 12 h and 30 h, whereas after 48 h, treated seeds were able to restore respiration to control levels. These results are in agreement with the in vivo ^31^P-NMR data, where an evident reduction in ATP content was observed after 24 h, which was compensated after 48 h of treatment. As previously, dry seeds contain only small amounts of ATP, but it is rapidly produced during cellular hydration in association with respiration [39,40,41]. Moreover, in cumarin-treated seeds, a higher value of vacuolar phosphate was observed after 48 h, suggesting that (i) the phosphate mobilisation from phosphate stored forms (i.e., phytate) was not affected by coumarin treatment, and (ii) phosphate transported to the cytosol and used by treated seeds was lower than the control, probably due to a delay in the reactivation of seed metabolic processes.

^31^P-NMR data, which joined to the previously described data to support a delay in respiration processes and a reduction in ATP production, were strongly supported by the untargeted metabolomic data. In fact, the pathway analysis highlighted an alteration of the TCA and glyoxylate cycle, the last pivotal in oilseeds (such as *R. sativus*) during reserve mobilisation [42]. In fact, most of the metabolites involved in those pathways, also identified by the random forest analysis as potential biomarkers (i.e., isocitric acid, malic acid, succinic acid, and fumaric acid), were significantly accumulated in treated seeds after 24 h of treatment. It is known that many key enzyme activities and products of the TCA cycle increase during early germination [29,43]; hence, our result suggest that the germinating seeds were not able to consume them for energy production because of reduced respiration. They then dropped after 48 h, confirming a potential recovery of the physiological processes (i.e., respiration, among others) in which they are involved.

Additionally, the majority of the identified amino acids, known to strongly accumulate during seed germination [29,44], significantly increased after 24 and 48 h of treatment, but in coumarin-treated samples, their content was lower than the control, further suggesting that the normal biochemical processes in germinating seeds were slowed down. The aspartate amino acid family (lysine, methionine, aspartic acid, among others) seems to be crucial for seed germination, and it has been observed that during this physiological process, aspartate is the most increased amino acid [45]. Additionally, lysine, an aspartate metabolism product, was proved to provide substrate for the TCA cycle since the manipulation of biosynthetic and catabolic enzymes involved in its metabolism has a major effect on the level of metabolites of the TCA cycle, suggesting a strict connection between lysine metabolism and the cellular energy metabolism [29,46,47]. In particular, it has been observed that transgenic lines, characterised by increased lysine synthesis during seed germination, presented a slowing down of this process, altering several metabolites connected to the TCA cycle [46,48]. These results suggest that the catabolism of the amino acids belonging to the aspartic amino acid family is an important contributor to the energy status of plants and the start of autotrophic-growth-associated processes during germination [46]. Interestingly, as observed in our experiments, where lysine was accumulated more than the control at 24 h and dropped after 48 h, the increase in lysine was also accompanied by a decrease in aspartic acid, phenylalanine, fumaric acid, malic acid, and succinic acid [46].

## 4. Materials and Methods

### 4.1. Plant Material, Treatment, and Germination Indexes

To allow the synchronisation of seed germination, the seeds of *Raphanus raphanistrum* subsp. *sativus* (L.) Domin were poured into a beaker filled with deionised water and incubated for one hour in a refrigerator at 4 °C to soften the integuments. Successively, the seeds were blotted on filter paper, and the teguments were removed using a razor blade. Shelled seeds were then transferred to Petri dishes (9 cm in Ø), with the bottom covered by a single layer of filter paper. The experiments were carried out using 10 seeds per Petri dish; the experiments were replicated 4 times and validated by repeating them twice.

To identify the dose that delayed but did not inhibit germination, a coumarin dose–response curve was built by pouring to each Petri dish 5 mL of aqueous solutions with different coumarin concentrations: 0, 25, 50, 100, 200, 400, and 800 µM.

Coumarin (99% in purity) was bought from Sigma-Aldrich, Italy (catalogue number: C4261-50G) and solubilised in deionised water.

The germination was monitored daily for 72 h by checking root protrusion (no differences in germination between 48 and 72 h were observed), and then a germination index (GT(%)), and the speed of germination (S) was calculated using the following formulas:

GT(%) = [(NT ∗ 100)/N] (NT, number of germinated seeds; N, number of seeds used in the bioassay);

S = (N1 ∗ 1) + (N2−N1) ∗ 1/2 (N1, N2, N3, ……Nn−1, Nn); the proportion of the germinated seed obtained at the first (1), the second (2), the third (3), (n−1), and (n) hours after sowing).

### 4.2. Ion Concentration in the Incubation Medium

The concentration of ions in the incubation medium was measured at 12 h, 24 h, 36 h, and 48 h using a Varian 820 ICP-MS (Varian, Inc., Palo Alto, CA, USA). A 2 mg L^−1^ aliquot of an internal standard solution (45Sc, 89Y, 159Tb) was added to both samples and the calibration curve to give a final concentration of 20 μg L^−1^. Typical analysis interferences were removed by using the collision–reaction interface of the ICP-MS with an H2 flow of 40 mL min^−1^.

### 4.3. Oxygen-Uptake Rate Evaluation

Oxygen-uptake rates were measured by using a Gilson differential respirometer IGRP 20 (Gilson Medical Electronics, Middleton, WI, USA). Seeds, previously incubated for different times in a flask, were transferred into the reaction vessels with 3 mL of the same solution previously used. The central well of each flask contained a fluted filter paper wetted with 0.2 mL of 1 M KOH. The rate of oxygen uptake was measured for 15 min at 26 °C in the dark after 30 min of equilibration.

### 4.4. Nuclear Magnetic Resonance Spectroscopy

The in vivo ^31^P-NMR spectra were recorded on a standard broad-band 10 mm probe on a Bruker AMX 600 spectrometer (Bruker Analytische Messtechnik, Ettlingen, Germany) equipped with TopSpin software, version 1.3. The ^31^P-NMR spectra were recorded at 242.9 MHz without lock, with a Waltz-based broad-band proton decoupling and a spectral window of 16 kHz.

Chemical shifts were measured relative to the signal from a glass capillary containing 33 mM methylenediphosphonate (MDP), which is at 18.5 ppm relative to the signal from 85% H_3_PO_4_. The experiments were carried out by packing the seedlings, previously incubated for 24 or 48 h in the absence or in the presence of 400 µM of coumarin, into a 10 mm-diameter NMR tube equipped with a perfusion system connected to a peristaltic pump in which the aerated, thermoregulated (26 °C) medium (1 mM MDP, 0.4 mM CaSO_4_, 1 mM MES-BTP (pH 6.1) ± 400 µM coumarin) flowed at 10 mL min^−1^. The spectra were determined using a 90° pulse and a recycle time of 1 s (fast acquisition conditions) or 6 s (to give fully relaxed resonance, except for vacuolar phosphate). Resonances were assigned according to Roberts et al. [49] and Kime et al. [50]. Metabolite concentrations in the tissue were determined according to Spickett et al. (1992) by comparing the resonance intensities with that of a glass capillary containing 33 mM MDP and previously calibrated against standard solutions. The areas of the ^31^P peaks were measured by the percentage volume of the tissue in the NMR tube [51]. Values of cytoplasmic pH (pHc) and vacuolar pH (pHv) were estimated from the chemical shift of Pi resonance after the construction of a standard titration curve [52].

### 4.5. Untargeted Metabolomic Analysis

To evaluate the effects of coumarin on *R. sativus* seeds’ metabolism during the germination process, shelled seeds were treated, as previously described, using coumarin at the concentration of 400 µM. Seeds were collected at different time points (T0 = 0 h; T1 = 6 h; T2 = 12 h; T3 = 24 h; T4 = 48 h), snapped frozen in liquid nitrogen (to quench the endogenous metabolism), and powdered, and 100 mg of plant material for each replicate was transferred into a 2 mL vial.

Extraction was completed by adding 1400 µL of methanol (at −20 °C) and vortexing for 10 s after the addition of 60 µL ribitol (0.2 mg mL^−1^ stock in ultrapure H_2_O) as an internal quantitative standard for the polar phase. Samples were transferred in a thermomixer at 70 °C and were shaken for 10 min (950 rpm) and then further centrifuged for 10 min at 11,000 g. The supernatants were collected and transferred to glass vials, where 750 µL CHCl3 (−20 °C) and 1500 µL ultrapure H_2_O (4 °C) were sequentially added. All the samples were vortexed for 10 s and then centrifuged for another 15 min at 2200 g. The upper polar phase (150 µL) for each replicate was collected, transferred to a 1.5 mL tube, and dried in a vacuum concentrator without heating. Then, 40 µL methoxyamine hydrochloride (20 mg/mL in pyridine) was added to the dried samples, which were immediately incubated for 2 h in a Thermomixer (950 rpm) at 37 °C. Methoxyamated samples were then silylated by adding 70 µL of MSTFA to the aliquots. Samples were further shaken for 30 min at 37 °C. Derivatised samples (110 µL) were then transferred into glass vials for GC/MS analysis.

### 4.6. GC-Quadrupole/MS Analysis

The derivatised extracts were injected into a MEGA-5MS capillary column (30 m × 0.25 mm × 0.25 µm equipped with 10 m of pre-column) using a gas chromatograph apparatus (Agilent 7890A GC) equipped with a single quadrupole mass spectrometer (Agilent 5975C). Injector and source were set at 250 °C and 260 °C temperatures, respectively. One µL of the sample was injected in splitless mode with a helium flow of 1 mL/min using the following programmed temperature: isothermal 5 min at 70 °C followed by a 5 °C/min ramp to 350 °C and a final 5 min heating at 330 °C. Mass spectra were recorded in electronic impact (EI) mode at 70 eV, scanning at 40–600 *m*/*z* range, scan time 0.2 s. The mass spectrometric solvent delay was settled as 9 min. Pooled samples that served as quality control (QCs), n-alkane standards, and blank solvents (pyridine) were injected at scheduled intervals for instrumental performance, tentative identification, and monitoring of shifts in retention indices (RI). Solvent blanks were run between samples, and each mass was checked against the blank run to exclude possible contamination sources.

### 4.7. GC/MS Data Analysis Using MS-DIAL

The MS- DIAL with open-source publically available EI spectra library was used for raw peaks extraction, and the data baseline filtering and calibration of the baseline, peak alignment, deconvolution analysis, peak identification, and integration of the peak height. The average peak width of 20 scans and a minimum peak height of 1000 amplitudes was applied for peak detection, and the sigma window value of 0.5, EI spectra cut-off of 5000 amplitudes was implemented for deconvolution. For peak identification, the retention time tolerance was set at 0.2 min, the *m*/*z* tolerance was 0.5 Da, the EI similarity cut-off was 60%, and the identification score cut-off was 80%. In the alignment-parameters-setting process, the retention time tolerance was 0.5 min, and the retention time factor was 0.5.

For MS-DIAL data annotations, we used publicly available libraries (both positive and negative) for compound identification based on the mass spectral pattern compared to EI spectral libraries such as the MSRI spectral libraries from Golm Metabolome Database [53] available from Max Planck Institute for Plant Physiology, Golm, Germany (http://gmd.mpimp-golm.mpg.de/, accessed on 2 March 2022), MassBank [54], MoNA (Mass Bank of North America, (http://mona.fiehnlab.ucdavis.edu/, accessed on 2 March 2022).

For relative quantification purposes, when we encountered multiply silylated (n-TMS) features of well-annotated metabolites, we maintained the major (higher in abundance) compounds and discarded minor compounds (lower in abundance) for consistent comparison across all samples.

Once the compounds and features were identified and annotated, the shared metabolites were only reported as quantified and confidently identified. For metabolite annotation and assignment of the EI-MS spectra, we followed the metabolomics standards initiative (MSI) guidelines for metabolite identification [55], i.e., Level 2: identification was based on the spectral database (match factor >80%) and Level 3: only compound groups were known, e.g., specific ions and RT regions of metabolites and Level 4: in silico annotation. The Level 4 identification of unknown EI-MS features that did not match the existing spectral libraries was carried out using MS-FINDER version 3.44 [56].

### 4.8. Statistical Analysis

Experiments were carried out using a randomised design with four replications for germination experiments and GC-MS-driven untargeted metabolomics (N = 4), and three replications for NMR, ionomic analysis, and respiration experiments (N = 3).

Germination, respiration parameters, NMR, and ionomic analysis were previously tested for normality and homogeneity of the variance and successively analysed through one-way ANOVA using the LSD test as post hoc (*p* ≤ 0.05).

Metabolomic data were analysed using the software Metaboanalyst 5.0 [25]. Metabolomics data were normalised using the internal standard and QCs for LOESS-based normalisation functions available in the MS-DIAL software for batch-correction procedures. The missing values of the Lowess normalised dataset were replaced with half of the minimum value found in the data set. Successively, data were Log2 transformed, and Pareto-scaled. Data were then classified through principal component analysis (PCA) to have an overview of the quality of the data acquisition step, while the PLS-DA was employed to identify the differential metabolites by calculating the corresponding variable importance in the projection (VIP value).

A within-subjects two-way ANOVA was used, and the significance threshold was defined as the corrected *p*-value < 0.05. The False Discovery Rate was chosen for multiple testing corrections. For ASCA, the leverage threshold and alpha threshold were set to be 0.8 and 0.05, respectively.

Enrichment and pathways analysis was carried out using the Metaboanalyst 5.0 tools and setting Arabidopsis thaliana as a metabolome reference database.

## 5. Conclusions

The potential use of coumarin as a potential botanical herbicide has been largely discussed in the bibliography, but still, no clear information has been reported concerning the primary target of this molecule since the majority of the experiments carried out on this molecule were completed using a long time of exposure.

Understanding the mode of action of natural compounds is challenging since it requires biochemical and physiological knowledge as well as the choice of the right concentration and time of exposure. To highlight the primary biochemical and physiological processes altered by exogenous molecules and to avoid observing side effects due to a cascade of biochemical and physiological responses, we decided to work with low effective concentrations monitoring the seed responses in a short period.

The results highlighted that coumarin was able to interact with membranes’ reorganisation, delaying them and reducing the production of ATP, as also supported by the pathway analysis and cell respiration. The NMR analysis supported the hypothesis that the concentration chosen was able to affect plant metabolism, maintaining, on the other hand, its viability, which is extremely important for studying natural compounds’ mode of action. In addition, coumarin treatment strongly affected the seed metabolome, the trend of which generally followed the control, but it was characterised by a lower level of metabolites. The metabolomic study also highlighted different behaviour of the amino acid lysine, whose content was significantly increased after 24 h of treatment. Interestingly such an increase is generally connected, as also observed in our experiment, to germination delay and alteration of TCA cycle metabolites. Although this is a preliminary screening that has allowed us to highlight the main biochemical and physiological targets and processes involved in coumarin phytotoxicity on seeds, further studies are required to study them in-depth to better understand coumarin’s mode of action.

This study further confirmed that coumarin is an extremely biologically active molecule representing a promising pharmacophore for the development of new agrochemicals with a low impact on the environment and human health. Moreover, its ability to delay germination in several species, reducing their fitness and competitiveness with crops, is in agreement with the agroecological perspective, which requires new agrochemicals that reduce weed pressure on crops without altering the field biodiversity.

## Figures and Tables

**Table 1:** Cytoplasmic and vacuolar pH of *R. sativus* seeds incubated for 24 and 48 h in the absence or in the presence of 400 µM of coumarin. The pH values were calculated from the chemical shift of Pi (inorganic phosphate) after the construction of a standard titration curve and are the average of three independent experiments.

Incubation Time (h)	Cytoplasmic pH		Vacuolar pH	
	Control	Coumarin	Control	Coumarin
24	7.74 ± 0.07 ^a^	7.77 ± 0.04 ^a^	5.92 ± 0.11 ^a^	6.08 ± 0.04 ^a^
48	7.63 ± 0.04 ^a^	7.57 ± 0.03 ^a^	5.73 ± 0.13 ^b^	5.97 ± 0.07 ^a^

Data are expressed as mean ± SD. Data were analysed through two-way ANOVA using the LSD test as post hoc (*p* ≤ 0.05). Different letters along the raws for cytoplasmatic pH and wacuolar pH indicate statistical differences among the treatments. N = 3.

**Table 2:** Result from “Pathway Analysis” (topology + enrichment analysis) carried out on the metabolite identified in *R. sativus* seeds during the germination process (T0–T2) in response to different coumarin doses (0 µM and 400 µM).

			T1		T2		
	Total Cmpd	Hits	Raw p	FDR	Raw p	FDR	Impact
Linoleic acid metabolism	4	1	2.60 × 10^−8^	1.34 × 10^−7^	9.34 × 10^−9^	1.81 × 10^−8^	1
Alanine aspartate and glutamate metabolism	22	7	3.92 × 10^−9^	4.05 × 10^−8^	2.78 × 10^−10^	1.01 × 10^−9^	0.58274
Isoquinoline alkaloid biosynthesis	6	1	8.49 × 10^−5^	0.00011	4.46 × 10^−9^	1.04 × 10^−8^	0.5
Phenylalanine metabolism	11	1	3.24 × 10^−5^	4.28 × 10^−5^	1.99 × 10^−6^	2.75 × 10^−6^	0.47059
Arginine biosynthesis	18	6	2.95 × 10^−6^	5.22 × 10^−6^	6.90 × 10^−9^	1.53 × 10^−8^	0.39224
Flavone and flavonol biosynthesis	10	1	0.000517	0.000616	1.45 × 10^−6^	2.05 × 10^−6^	0.35
beta-Alanine metabolism	18	3	3.35 × 10^−11^	1.04 × 10^−9^	4.96 × 10^−10^	1.62 × 10^−9^	0.3254
Glycine serine and threonine metabolism	33	5	3.18 × 10^−6^	5.47 × 10^−6^	1.21 × 10^−10^	4.99 × 10^−10^	0.3242
Arginine and proline metabolism	34	6	2.15 × 10^−6^	3.92 × 10^−6^	1.80 × 10^−10^	6.98 × 10^−10^	0.31071
Galactose metabolism	27	7	2.05 × 10^−5^	2.77 × 10^−5^	1.06 × 10^−10^	4.69 × 10^−10^	0.28841
Glyo×ylate and dicarbo×ylate metabolism	29	5	1.97 × 10^−9^	2.44 × 10^−8^	1.82 × 10^−12^	2.26 × 10^−11^	0.21995
Tyrosine metabolism	16	3	3.22 × 10^−8^	1.43 × 10^−7^	1.81 × 10^−8^	3.12 × 10^−8^	0.21622
Cysteine and methionine metabolism	46	5	2.83 × 10^−11^	1.04 × 10^−9^	2.93 × 10^−9^	7.25 × 10^−9^	0.19893
Starch and sucrose metabolism	22	2	1.24 × 10^−6^	2.32 × 10^−6^	3.82 × 10^−8^	5.90 × 10^−8^	0.17067
Sulphur metabolism	15	5	1.86 × 10^−5^	2.56 × 10^−5^	3.90 × 10^−8^	5.90 × 10^−8^	0.16851
Butanoate metabolism	17	3	1.13 × 10^−8^	8.53 × 10^−8^	6.70 × 10^−13^	1.64 × 10^−11^	0.13636
Stilbenoid diarylheptanoid and gingerol biosynthesis	8	1	//	//	0.024158	0.024554	0.13235
Phenylpropanoid biosynthesis	46	4	//	//	0.00847	0.009054	0.13199
Flavonoid biosynthesis	47	3	//	//	0.004188	0.004556	0.12717
Tryptophan metabolism	28	2	0.001924	0.002168	2.57 × 10^−9^	6.64 × 10^−9^	0.12037
Pantothenate and CoA biosynthesis	23	3	3.06 × 10^−8^	1.43 × 10^−7^	4.96 × 10^−11^	2.56 × 10^−10^	0.11663
Citrate cycle (TCA cycle)	20	3	4.97 × 10^−7^	1.14 × 10^−6^	5.50 × 10^−11^	2.62 × 10^−10^	0.11468
Aminoacyl-tRNA biosynthesis	46	14	7.07 × 10^−7^	1.51 × 10^−6^	9.43 × 10^−13^	1.64 × 10^−11^	0.11111
Amino sugar and nucleotide sugar metabolism	50	2	1.24 × 10^−8^	8.53 × 10^−8^	9.03 × 10^−10^	2.64 × 10^−9^	0.10791
Inositol phosphate metabolism	28	1	2.12 × 10^−7^	6.26 × 10^−7^	3.09 × 10^−11^	1.74 × 10^−10^	0.10251

Total Cmpd: the total number of compounds in the pathway; Hits: the matched number from the uploaded data; Raw p: the original *p* value, FDR: the false-discovery rate applied to the nominal *p*-values to control for false-positive findings; Impact: the pathway impact value calculated from pathway topology analysis. Only pathways with an impact higher than 0.1 were reported. For the full list of the significantly altered pathways, refer to Supplementary Table S1.

## Data Availability

The data presented in this study are available on request from the corresponding author.

## Supplementary Materials

The following supporting information can be downloaded at: https://www.mdpi.com/article/10.3390/plants11070843/s1, Figure S1: MS-DIAL PCA score plot of annotated and unknown compounds; Figure S2: Pairwise score plot for top 5 components of the PLS-DA; Figure S3: Cross-validation (a) and permutation test (b) used to validate the PLS-DA model’s robustness. Table S1: Metabolomic dataset and statistical analyses.
