# Supplementary figures and images for: The Delay of Raphanus raphanistrum subsp. sativus (L.) Domin Seed Germination Induced by Coumarin Is Mediated by a Lower Ability to Sustain the Energetic Metabolism
Source: Plants (Basel). 2022 Mar 22;11(7):843. doi: 10.3390/plants11070843 (PMC9002777; doi:10.3390/plants11070843)

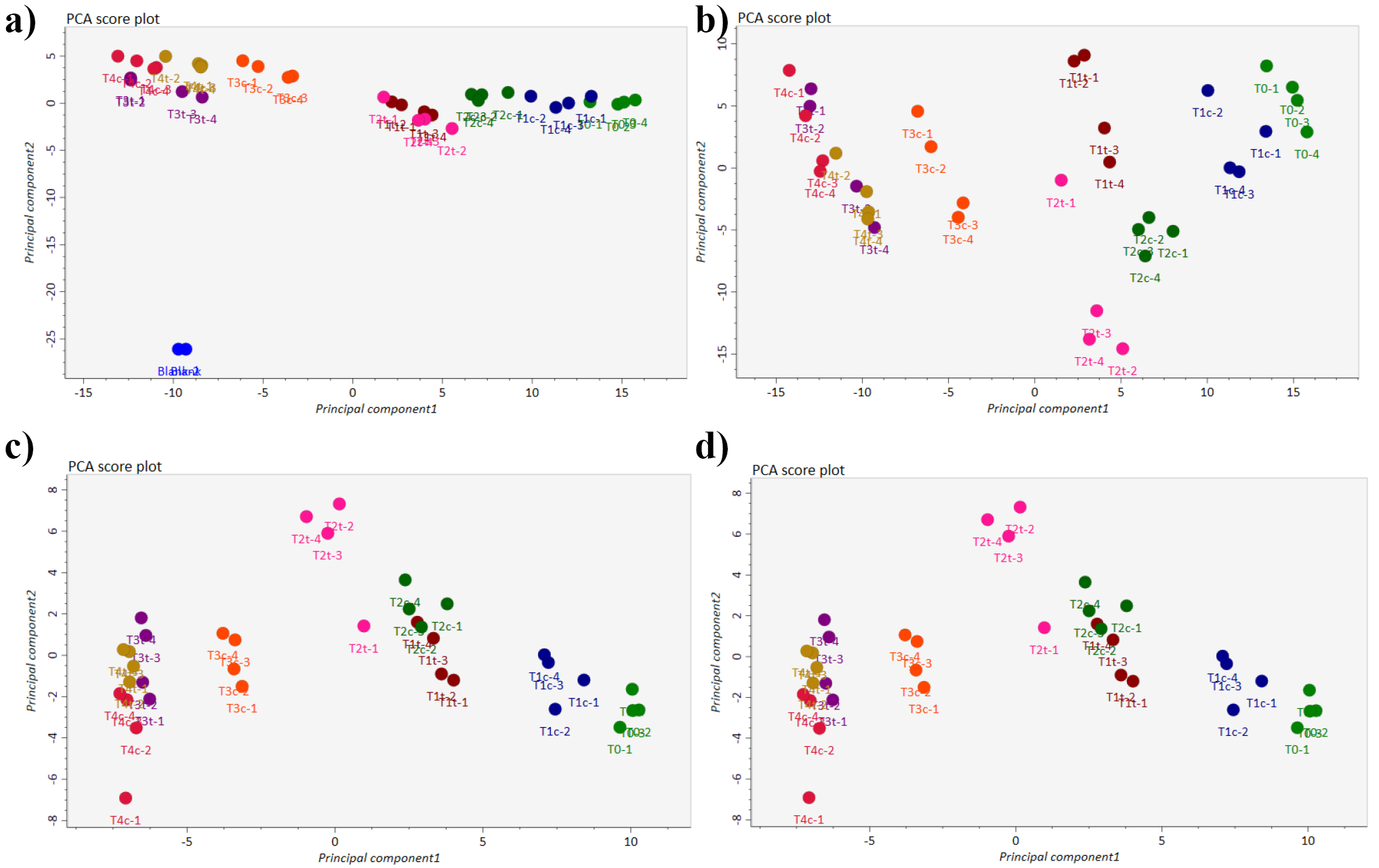

Supplement: Supplementary file 1 [file plants-11-00843-s001.zip › Supplementary Figure. S1.tif]

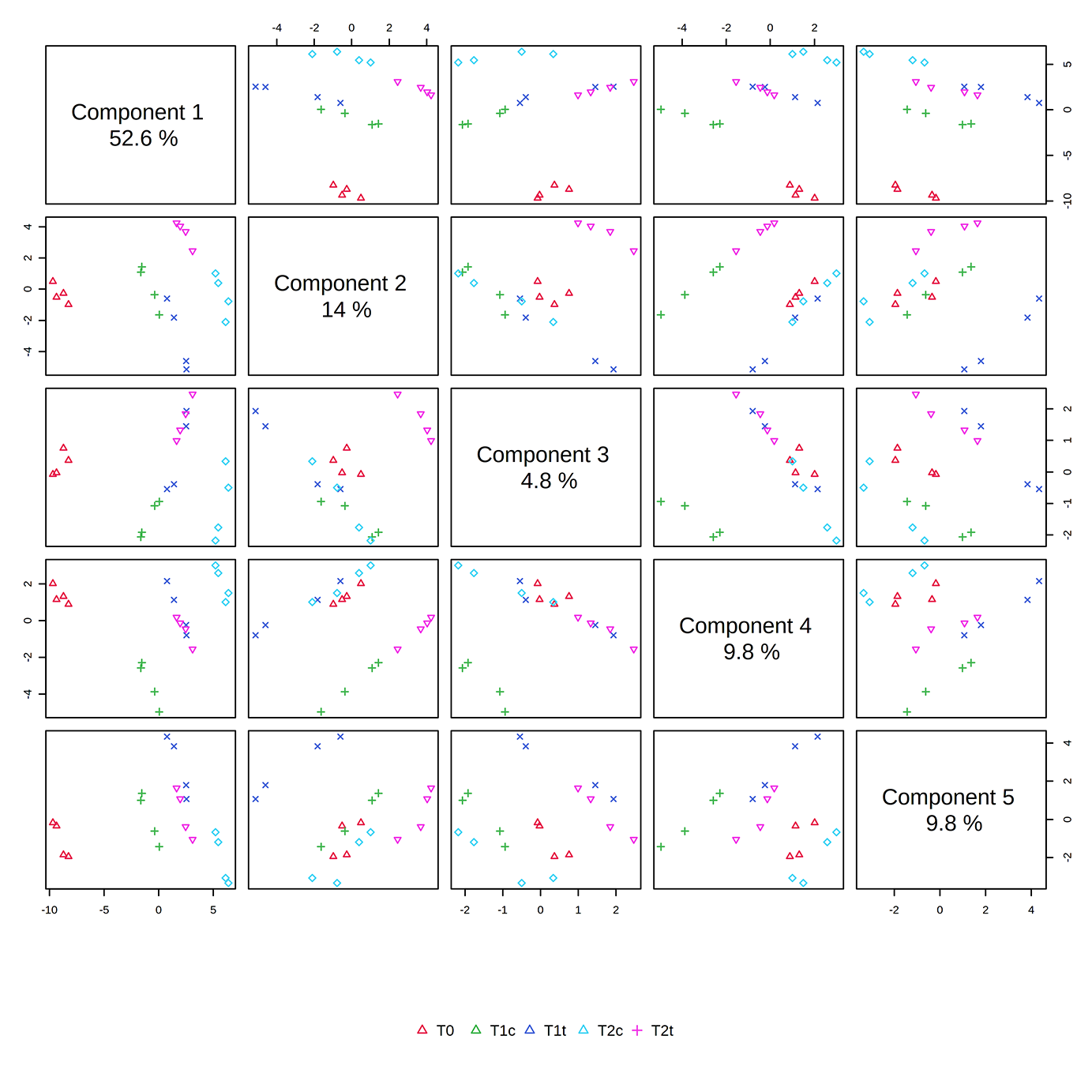

Supplement: Supplementary file 1 [file plants-11-00843-s001.zip › Supplementary Figure. S2.tif]

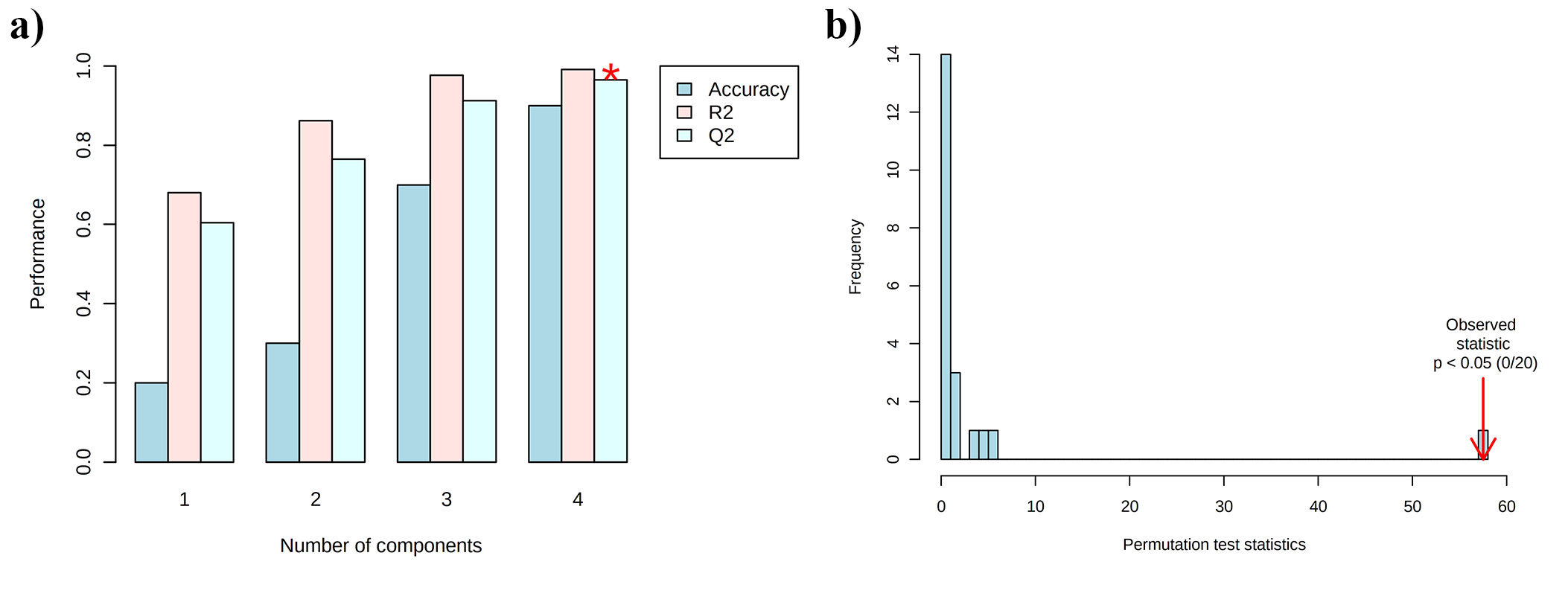

Supplement: Supplementary file 1 [file plants-11-00843-s001.zip › Supplementary Figure. S3.tif]
